# How patients in general practice voice and value their gut feelings about health: a qualitative interview study

**DOI:** 10.3399/BJGP.2022.0427

**Published:** 2023-08-22

**Authors:** Erik Stolper, Ulricke M Schuck, Antoinet Hoekman, Elena Shvarts, MA Loes van Bokhoven, Geert J Dinant, Paul Van Royen, Margje WJ van de Wiel

**Affiliations:** CAPHRI School for Public Health and Primary Care, University of Maastricht, Maastricht, the Netherlands; Faculty of Medicine and Health Sciences, Department of Family Medicine and Population Health, University of Antwerp, Antwerp, Belgium.; CAPHRI School for Public Health and Primary Care, University of Maastricht, Maastricht, the Netherlands.; Department of Work and Social Psychology, Faculty of Psychology and Neuroscience, Maastricht University, Maastricht, the Netherlands.; Faculty of Medicine and Health Sciences, Department of Family Medicine and Population Health, University of Antwerp, Antwerp, Belgium.; CAPHRI School for Public Health and Primary Care, University of Maastricht, Maastricht, the Netherlands.; CAPHRI School for Public Health and Primary Care, University of Maastricht, Maastricht, the Netherlands.; Faculty of Medicine and Health Sciences, Department of Family Medicine and Population Health, University of Antwerp, Antwerp, Belgium.; Department of Work and Social Psychology, Faculty of Psychology and Neuroscience, Maastricht University, Maastricht, the Netherlands.

**Keywords:** clinical reasoning, intuition of patients, mothers’ instinct, patients’ language, primary care, qualitative research

## Abstract

**Background:**

GPs consider their gut feelings a valuable tool in clinical reasoning. Research suggests patients’ gut feelings may be a useful contribution to that process. Describing these feelings more precisely could improve primary care professionals’ (PCPs) recognition of patients’ gut feelings and insight into the underlying reasons. These descriptions would also enable a thorough examination of the validity of patients’ gut feelings and their contribution to professionals’ clinical reasoning.

**Aim:**

To gather the words and phrases that patients or their relatives use to share their gut feelings with primary care professionals and what they convey and imply.

**Design and setting:**

Qualitative study of Dutch and Belgian patients visiting an out-of-hours GP service or a GP’s office.

**Method:**

Face-to-face semi-structured interviews were carried out with 47 patients. Interviews were coded using a descriptive content analysis in an iterative process until data sufficiency.

**Results:**

Patients or their relatives expressed their gut feelings by using words relating to trusting or not trusting the situation, or to changes in normal patterns. Their gut feelings are most often felt as a sense of alarm. In general, patients experiencing a sense of alarm, particularly mothers of sick children, were convinced that something was wrong and had often learned to trust their gut feeling. A gut feeling was the main reason to contact a PCP. Patients generally felt that their gut feelings were taken seriously.

**Conclusion:**

The findings of this study provide an insight into how patients and relatives may express their gut feelings about their own or their relative’s health and how they share these feelings with healthcare professionals. This may help clinicians improve their recognition of patients’ gut feelings, being particularly alert to a patient or relative using phrases that relate to feelings of not trusting a situation, things seeming wrong or different from normal, and experiencing a sense of alarm. Further research should be carried out into the validity of patients’ gut feelings.

## INTRODUCTION

GPs consider their gut feelings regarding a patient’s health a valuable tool in clinical reasoning, particularly in situations of diagnostic uncertainty.^1^ These gut feelings can be regarded as a specific kind of intuitive feeling usually confined to prognostic assessments of the patient’s situation and often accompanied by bodily sensations.^2^ A sense of alarm activates the diagnostic process by stimulating a GP to formulate and weigh up working hypotheses that might involve a serious outcome.^3^ A sense of reassurance means that a GP feels secure about the further management and course of a patient’s problem, even though they may not yet be certain about the diagnosis: ‘everything fits in’.^3^ GPs’ gut feelings have been proven to be measurable using a questionnaire (see www.gutfeelingsingeneralpractice.eu).^4^^–^^7^

It is evident that patients also have gut feelings concerning their health, as the cognitive decision-making process based on knowledge and experience is similar for physicians and patients.^8^ However, applying the definitions of GPs’ gut feelings to those of patients would be a mistake. Patients have different, less widely developed, and often less accurate knowledge about health and diseases than physicians. On the other hand, patients have experiential knowledge about their own health and body, which may signal any changes. Although they may struggle to verbalise these signals, they use this knowledge in their decision to consult their GP.^9^ In situations where medical evidence is lacking, incomplete, or inconclusive, this knowledge might support physicians’ clinical reasoning, especially in complex cases.^9^^,^^10^ Even when a patient’s intuition is mistaken, exploring their health beliefs and how these relate to their decision-making process might increase physicians’ insights and influence their management.^9^

Dutch and Belgian primary care professionals acknowledge their patients’ gut feelings and regularly use them in their communication with patients and in their clinical reasoning.^11^ They are able to describe some phrases and expressions that patients use to voice their gut feelings. Clinicians weigh the value of the patients’ gut feelings against their own judgement, which may result in reconsidering their diagnostic hypotheses and decision making. Additionally, medical disciplinary tribunals in the Netherlands consider patients’ gut feelings about their health to be a valuable part of a doctor’s diagnostics, which should make them review their clinical reasoning.^12^

**Table:** How this fits in

Primary care professionals acknowledge the usefulness of patients’ gut feelings in their clinical reasoning. However, the phrases and expressions that patients use to voice their gut feelings and how they share them with professionals are not precisely known. The results of this study may help clinicians improve their recognition of patients’ gut feelings and insight into the underlying reasons, as well as enable further research into their validity.

Almost all studies in the literature confirm the value of patients’ gut feelings. Callers to an out-of-hours GP service in Denmark proved to be able to quantify their degree of worry.^13^ This degree of worry was higher when the cause of the illness was unclear, and the consequences were uncertain.^14^ A qualitative study found that patients’ gut feelings about having cancer could be an important reason for further diagnostics.^15^ Parents’ feelings that ‘this illness is different from previous illnesses’ had a high odds ratio (36.3, 95% confidence interval [CI] = 12.3 to 107) for serious infections among children in family practice and influenced GPs’ gut feelings and decision making.^16^^,^^17^ In family practice, changes in the behaviour of their febrile child were a major reason for parents to seek care.^18^ Parents visiting the emergency department or a paediatric ward mentioned that the normal behaviour and physical features of their child represented a frame of reference when judging disease severity.^19^ Abnormalities might act as a kind of alarm, for instance, a feeling that there was something wrong with their child based on parental instinct.^19^ A study in a tertiary hospital, however, could not confirm the positive predictive value of parents’ concern.^20^

Patients’ gut feelings about their health might therefore be valuable for medical professionals’ clinical reasoning.^9^ To improve the professionals’ recognition and understanding of a patient’s gut feeling, it would be useful to describe these gut feelings more precisely. Additionally, such descriptions would enable examination of the validity of patients’ gut feelings, and how they may contribute to primary care professionals’ clinical reasoning.^9^ The objective of this study was to gather the words and phrases that patients or their relatives use to communicate their gut feelings to primary care professionals, and what these convey and imply. In addition, the study aimed to examine whether, in the patients’ perception, medical professionals understand their gut feelings and take them seriously. A longer-term aim of the study is to create a short questionnaire for measuring patients’ gut feelings.

## METHOD

### Participants

Interviews were conducted with 39 Dutch and eight Belgian patients living in urban or rural areas who visited an out-of-hours GP service (*n* = 29) or their GP during office hours (*n* = 18). The national language in the Netherlands and Belgium is Dutch. In almost all cases patients were interviewed before seeing their physician. Informed consent was obtained for every participant.

### Data collection

Interviews were held by one or two of the authors. Interviews (*n* = 47) were held face to face, using semi-structured interview guides for adults and for parents of ill children (Supplementary Box S1), and were audio-recorded.^21^ The questions in the guides were based on previous studies.^2^^,^^11^ Interviews focused on the perceived health situation of the patients interviewed, as well as the perceptions of their partners, if present, or their parents in the case of children <18 years. Participants’ underlying beliefs and feelings, and how these feelings were verbalised, were also examined. The interviews lasted an average of 16 minutes (range 4–35 minutes).

### Procedure

The study began in January 2017 and finished in February 2020 with interviews carried out with Dutch patients visiting an out-of-hours GP service in one city. Well-briefed triage nurses selected patients who rejected self-care advice or expressed a gut feeling when calling the out-of-hours desk. This selection procedure yielded eight patients in 24 hours. The study continued with patients being randomly asked to participate when they checked in at the out-of-hours desk, in another city. Most patients (*n* = 17 out of 20) accepted the invitation. The same random procedure was followed when selecting and interviewing Belgian patients who visited an out-of-hours GP service in a Belgium city (*n* = 4). In 2019, after a first analysis of the data, the research was broadened to patients in another setting using the same selection procedure, leading to an additional 14 Dutch and four Belgian patients visiting their GP clinic during office hours being interviewed. In total, 47 patients were included in the study (34 adults and 13 children). Relevant information from the interviews about gut feelings experienced in the past was also included (Tables 1 and 2).

**Table 1.** Characteristics of and information provided by interviewed Dutch and Belgian patients visiting an out-of-hours GP service

**Interview number**	**Duration of interview (min)**	**Adult(s)**	**Parent(s)**	**Current information^a^**	**Retrospective information**	**Country**	**Before or after consulting GP**	**Invited by**
1	7		✓	✓		Netherlands	After	Triage nurse
2	11		✓	✓	✓ (about adult)	Netherlands	After	GP
3	10		✓	✓	✓ (about adult)	Netherlands	Before	Triage nurse
4	14	✓		✓	✓ (about child)	Netherlands	Before	Triage nurse
5	11	✓		✓		Netherlands	Before	Triage nurse
6	6	✓		✓		Netherlands	Before	Triage nurse
7	10	✓		✓		Netherlands	After	GP
8	16		✓	✓		Netherlands	Before	Triage nurse
9	13	✓		✓		Netherlands	Before	At random
10	14	✓		✓		Netherlands	Before	At random
11	19	✓		✓	✓ (about adult)	Netherlands	Before	At random
12	16	✓		✓	✓ (about adult)	Netherlands	Before	At random
13	26		✓	✓	✓ (about child)	Netherlands	Before	At random
14	14	✓		✓		Netherlands	Before	At random
15	11		✓	✓		Netherlands	Before	At random
16	10	✓		✓		Netherlands	Before	At random
17	10		✓	✓	✓ (about adult)	Netherlands	Before	At random
18	9	✓		✓		Netherlands	Before	At random
19	9	✓		✓		Netherlands	Before	At random
20	12	✓		✓	✓ (about adult)	Netherlands	Before	At random
21	14	✓		✓	✓ (about child)	Netherlands	Before	At random
22	17		✓	✓	✓ (about child)	Netherlands	Before	At random
23	15		✓	✓	✓ (about child)	Netherlands	Before	At random
24	25	✓		✓	✓ (about child)	Netherlands	Before	At random
25	4	✓		✓		Netherlands	Before	At random
26	15	✓		✓		Belgium	Before	At random
27	29	✓		✓	✓	Belgium	After	At random
28	34	✓		✓	✓	Belgium	Before	At random
29	18	✓		✓		Belgium	After	At random

a *Current information refers to information from that moment, that is, the reason for the encounter.*

**Table 2.** Characteristics of and information provided by interviewed Dutch and Belgian patients visiting their GP during office hours

**Interview number**	**Duration of interview (min)**	**Adult(s)**	**Parent(s)**	**Current information^a^**	**Retrospective information**	**Country**	**Before or after consulting GP**	**Invited by**
30	10		✓	✓	✓ (about child)	Netherlands	Before	Practice nurse
31	16	✓		✓	✓ (about partner)	Netherlands	Before	Practice nurse
32	18	✓		✓		Netherlands	Before	Practice nurse
33	17	✓		✓	✓ (about partner)	Netherlands	Before	Practice nurse
34	12	✓		✓	✓ (about partner)	Netherlands	Before	Practice nurse
35	14		✓	✓	✓ (about child)	Netherlands	Before	Practice nurse
36	12		✓	✓	✓ (about child)	Netherlands	Before	Practice nurse
37	17	✓		✓		Netherlands	Before	Practice nurse
38	19	✓		✓		Netherlands	Before	Practice nurse
39	17	✓		✓		Netherlands	Before	Practice nurse
40	15	✓		✓		Netherlands	Before	Practice nurse
41	21	✓		✓	✓ (about adult)	Netherlands	Before	Practice nurse
42	10	✓		✓		Netherlands	Before	Practice nurse
43	11		✓	✓	✓ (about child)	Netherlands	Before	Practice nurse
44	19	✓		✓	✓ (about child)	Belgium	Before	Reception staff
45	20	✓				Belgium	Before	Reception staff
45	35	✓		✓		Belgium	Before	Reception staff
47	30	✓		✓	✓ (about child)	Belgium	After	Reception staff

a *Current information refers to information from that moment, that is, the reason for the encounter.*

### Analysis

All verbatim texts were coded using a descriptive content analysis approach.^21^^,^^22^ A coding book was composed, using an iterative process starting with open coding and then searching for the main emergent themes addressed in the interviews. Three authors coded the data and reached consensus during the process by double coding and by discussing all codes and disagreements with two other authors. The data were sufficiently rich to answer all research questions, therefore data sufficiency was reached.^23^ As no differences were found in how the Dutch and Belgian patients expressed their gut feelings, it was decided not to include more Belgian patients. The report of this study is in line with the COREQ criteria.^24^ All data were anonymously gathered; therefore, a member check was not possible.

## RESULTS

**Figure 1.**
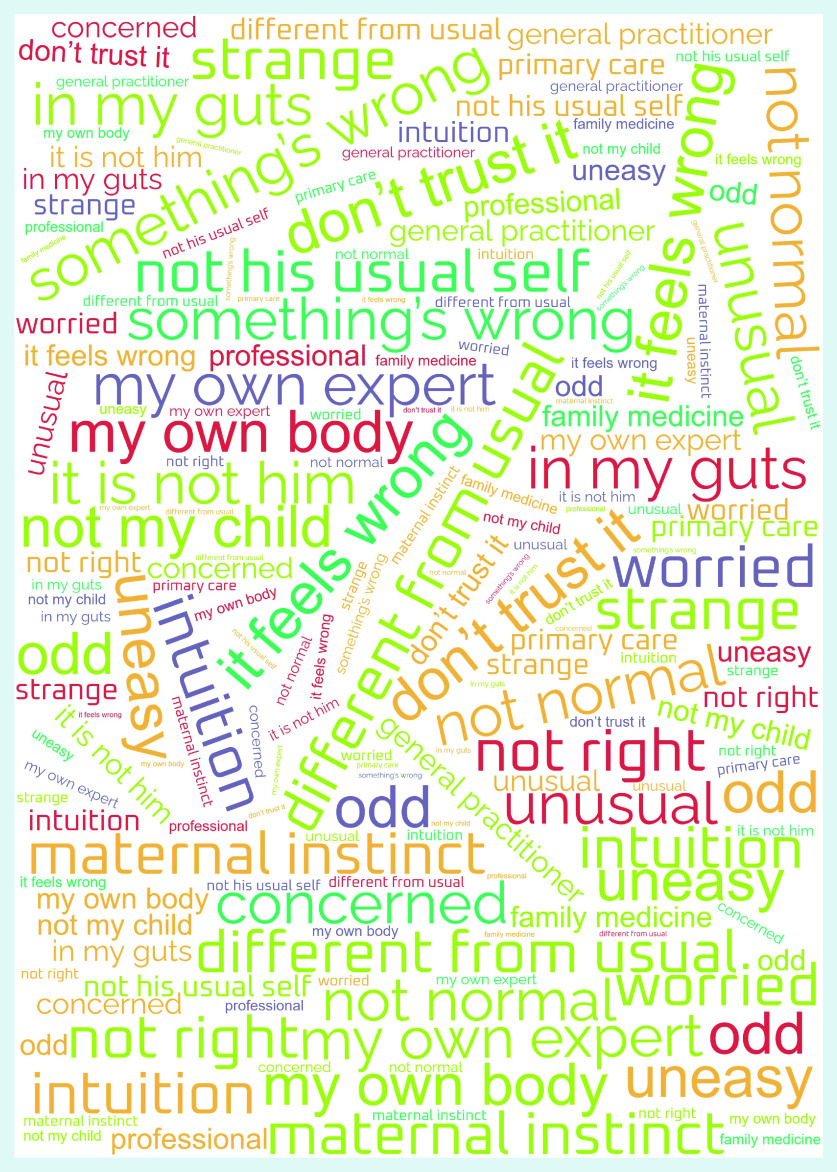
*Word cloud representing patients’ and relatives’ gut feelings.*

In general, it was not always easy for patients or their relatives to talk about their feelings of worry. A patient might initially appear not to be worried about symptoms, and then later in the interview spontaneously admit to being concerned. The content analysis resulted in four main themes, with a different emphasis for adult patients and their relatives, and for parents of sick children — distrust and pattern changes, confidence in one’s own gut feelings, anxiety and uncertainty, and inducing action; and a common theme for all participants — sharing gut feelings with a professional. Participants’ quotes illustrate the themes and are provided with an interview number (IN) (Tables 1 and 2). As the results did not differ between those patients visiting an out-of-hours GP service and those attending during GPs’ office hours, they are reported together. Figure 1 illustrates the words and/or phrases used by participants when describing their gut feelings in the form of a word cloud.

### Distrust and pattern changes

#### Adult patients and their relatives

Patients or their relatives who expressed their gut feeling used words relating to trusting or not trusting the situation or to any changes from normal, familiar patterns:

*‘ I don’t know what’s the matter with me, but something’s not right. ’*

(IN 4)

*‘ That something’s really going on, is not right, something’s not right. ’*

(IN 29)

*‘ It’s different from the usual. ’*

(IN 43)

*‘ I think it’s strange. ’*

(IN 31)

*‘ I’m worried. ’*

(IN 26)

*‘ I’m concerned. ’*

(IN 27)

Patients may experience a gut feeling in their body:

*‘ It’s a feeling in my guts. ’*

(IN 29)

There were also patients expressing a sense of reassurance:

*‘ I’m feeling fine. ’*

(IN 20)

Nevertheless, these patients could also show a kind of doubt:

*‘ It’s nothing very serious, fortunately … or I hope so anyway. ’*

(IN 20)

#### Parents of sick children

Parents also expressed their gut feeling using words relating to distrusting their children’s health and/or to any changes from normal, familiar behaviour patterns of their sick children. Many participants used words such as: *‘ it feels wrong* ’, *‘it’s just not going well at all’*, *‘ I’m really worried ’*, *‘ I can feel when it’s okay or not ’*, *‘ this is not normal* ’, or *‘ I’m unhappy about it ’.*

Parents mostly based their gut feeling on specific knowledge about their child:

*‘ It was not him, not like his usual self. We thought it was odd, him being like that. This was not right for him. We can read him very well. ’*

(IN 3)

*‘ I didn’t trust it, it didn’t feel right. It was my child, but then again not my child. I don’t know how to explain it. His eyes were unusual. His eyes were glazed. It was as if he looked right through you, and whatever you said to him he didn’t seem to get. Though his eating, drinking, and playing were normal. ’*

(IN 13)

When phrasing their gut feeling, parents did not often mention somatic symptoms such as high fever or cough.

Some parents found it difficult to explain where a gut feeling came from, while some referred to it as a maternal instinct, a matter of knowing for certain without knowing why:

*‘ I was really very worried … when I looked into the cradle and thought “yes, there really is something wrong with you” … I don’t know where that came from … something’s really wrong. ’*

(IN 24)

*‘ I think it’s a mother’s feeling … that as a mother you learn how to notice how the child responds … you know how to judge that. ’*

(IN 13)

*‘ A maternal instinct, that’s the word … you just know something is wrong. ’*

(IN 47)

In a few cases, a paternal instinct was mentioned.

Parents could also feel a gut feeling in the body:

*‘ An uneasy feeling … in your body, in your throat, something like, tension … a sixth sense. ’*

(IN 21)

### Confidence in one’s own gut feelings

#### Adult patients and their relatives

Most participants with a gut feeling were convinced that there was something wrong, and they often trusted their feeling:

*‘ I know very well if there’s something really wrong with me. ’*

(IN 3)

*‘Look, there’s only one person who knows my body best, and that’s me … I’m the expert on my own body.’*

(IN 47)

Some participants felt that they, or their physicians, had ignored their gut feeling with serious consequences. These experiences strengthened the confidence in their intuitive assessments.

Some participants, however, mistrusted their gut feeling because of negative experiences in the past:

*‘ I’m not going to just go by my gut feeling alone. ’*

(IN 29)

*‘ I’ve too often had it that I worried about all kinds of stuff* [in my partner] *and it was just nothing. I can get a feeling, but I try to base it on something. And if I can’t, I’ll ignore it. ’*

(IN 33)

#### Parents of sick children

In general, parents, specifically mothers, trusted their gut feelings or had learned to trust them:

*‘ When they were little,* [I didn’t] *always* [trust those feelings]*, but now I do … I think you have to learn that, to evaluate them … especially with your first child. ’*

(IN 13)

*‘ I’ve learned that your feeling rarely lets you down. ’*

(IN 9)

Past experiences of themselves or their physician wrongly ignoring a sense of alarm enhanced the trust in their gut feeling:

*‘ Since in the past I’ve sometimes kept it to myself, with serious consequences … that* [confidence] *has grown over the years; just daring to say things. ’*

(IN 32)

*‘ If I don’t trust it, I need to go on banging my fist on the table anyway. ’*

(IN 2)

One participant did not fully trust her gut feeling:

*‘ I’m often needlessly worried as there’s nothing wrong. ’*

(IN 15)

One mother mentioned that she was a very rational individual but still trusted her gut feeling:

*‘ It* [a mother’s feeling] *didn’t let me down … It’s not that if you’re rational it means you don’t have a mother’s intuition. ’*

(IN 46)

### Anxiety and uncertainty

#### Adult patients and their relatives

When participants were unable to explain their symptoms, their anxiety about the future and the sense of losing control over their lives increased, as well as their uncertainty about how to deal with the symptoms. This happened, for instance, when the complaints (or their number) or the course of the illness did not fit in with the explanation or diagnosis given by a primary care professional, or after finding alarming information on the internet:

*‘ My feeling was actually one of uncertainty, especially as you don’t know exactly where you’re heading, what’s going to happen to you … really very uncertain. ’*

(IN 27)

Uncertainty was less well tolerated when it concerned symptoms in vital body areas such as the chest or the head:

*‘ You can just about guess, based on your intuition, what the problem is and where it’s located. If the problem is here* [points at leg]*, you then say, “ Well, it’ll probably be gone tomorrow or the day after.” But if the problem is somewhere around here* [points at chest]*, for instance, then you might say, “It’s time for rapid action*.*”* ’

(IN 11)

A partner explained that she was better able to judge the patient’s situation just because of the distance:

*‘ If you’re looking at the patient, you’re not preoccupied with the actual pain and stress, and you’re better able to observe them and say, like, “I know what you’re normally like. I’ve known you long enough, and now there’s something different about you.”’*

(IN 29)

Conversely, another partner mentioned that she knew herself better:

*‘ If it’s about myself I tend to think “Oh it will pass” or “Well, I’d better get this looked at.” You know yourself better, you yourself feel, you feel what you feel … I can’t feel what he* [the partner] *feels. ’*

(IN 10)

#### Parents of sick children

Parents of sick children may experience mixed feelings of anxiety, uncertainty, sympathy, responsibility, and powerlessness:

*‘ So I was right to be worried. I don’t know where it came from … something’s really wrong. ’*

(IN 24)

*‘ It’s more the feeling that he* [my son] *needs to be able to trust that if something’s wrong, that I’ll deal with it, that I’ll take care of him. ’*

(IN 1)

*‘ A kind of powerlessness. There’s nothing you can do, and he’s just lying there. ’*

(IN 23)

Some parents did not see a difference between their gut feelings about themselves and those about their children:

*‘ I can read both myself and both of my children reasonably well. ’*

(IN 3)

Other parents emphasised the differences:

*‘ Those are two different things. Not that I’m less valuable, but the feeling is different. ’*

(IN 13)

They took a gut feeling about their child more seriously or mentioned that it more quickly led to some kind of action:

*‘ To me, it’s a different feeling. I think the feeling towards them is stronger than towards myself. ’*

(IN 22)

*‘ When it’s about my children, I take action more quickly, as I know they’re a bit more vulnerable. ’*

(IN 17)

One mother said that the responsibility for her child outweighed the responsibility for her partner:

*‘ I’m responsible for them* [child]*. My husband is a grown man. ’*

(IN 32)

### Inducing action

#### Adult patients and their relatives

A gut feeling was often the reason for patients to contact their GP, to clarify their health situation, or to be reassured:

*‘ Just to know where I’m at, to be sure whether it’s something or nothing.* ’

(IN 39)

‘ *I first listen to the doctor, and then I draw my own conclusion. ’*

(IN 27)

*‘ If I* [the partner] *were you, I’d go see the doctor, as you’re not your usual self. ’*

(IN 46)

Some patients felt a sense of urgency and forced their GP to act:

*‘ I’ll then take action, like* [my partner] *is not okay. I can be really forceful then. ’*

(IN 10)

Other patients ignored their worries because they were not able to cope with them emotionally or because of a lack of time:

*‘ I’m not keen on tackling the next* [problem]*. So I prefer to pretend that it* [the feeling] *isn’t there. ’*

(IN 11)

#### Parents of sick children

Parents’ gut feelings mostly led them to search for information on the internet or to contact their GP for advice or a consultation:

*‘ If I get the intuition, or at least the idea that it feels wrong, I pick up the phone immediately. ’*

(IN 21)

*‘ If I’m really worried as I think my child’s ill, I’ll take the child to our family doctor. ’*

(IN 26)

One mother with a gut feeling did not accept the explanation that the GP on duty had given to her partner about their ill child and phoned the triage nurse again:

*‘ I rang them again as I wasn’t happy about the way it was handled. I wanted to see some action. ’*

(IN 32)

### Sharing gut feelings with a professional

After the interview had finished and the underlying aim of the study was explained in the debriefing, participants often said that they appreciated the opportunity to tell the story of their current health, often linking it to experiences in the past:

*‘ During a normal consultation you don’t often get asked about the full picture, say about the journey you’re on, with your body and your illness. ’*

(IN 9)

#### Nurses

Patients were less inclined to share their gut feelings with triage or practice nurses than with their GP. Some patients had not expressed their feelings of worry or had downplayed them, while others had voiced their gut feelings clearly, and sometimes discussed them in a shared decision-making process:

*‘ I tried to describe a bit what the situation was like and what I had observed, how I was reading the situation, how he* [my child] *responded to certain things, so I think the receptionist* [triage nurse] *could evaluate whether it was better to get over there. ’*

(IN 15)

*‘ When I’m really worried, I’ll then say, “Well, I’m not easy about it.” In this case it was more, like, “There’s something wrong and it might be a good idea to have it looked at.”’*

(IN 21)

#### GPs

Most participants said that they would share their gut feelings with their GP or had shared them in the past, using the expressions reported above:

*‘ Yes, I would say that … I would be honest about it, very direct, about what I feel, like: “I’m really worried … it’s not okay at all.”’*

(IN 36)

Participants expected primary care professionals to take their gut feelings seriously by listening, examining, and offering clear explanations, which was usually found to be the case:

*‘ My GP trusted my feelings. And that felt just fine. ’*

(IN 13)

‘ *She* [the GP] *said “Sometimes you just have to fight like a lioness for your child.” She said that literally.* ’

(IN 32)

*‘ It’s good if at any rate the GP says, like, “I can’t find anything worrying now, but if you see things again, try to record it or phone the moment something happens.”’*

(IN 24)

Some Belgian participants were more reticent about communicating their gut feelings to their GP, as they tended to respect their GP’s expertise, and did not want to bother them:

*‘ Well, you know, you’re sitting in front of an expert, a doctor, a professional, and that makes it a bit scary to say that* [something is not correct]*. ’*

(IN 29)

*‘ This kind of “That’s what I’ve got, or I fear this and I think that” … I can imagine that some doctors get a bit annoyed about that. ’*

(IN 46)

## DISCUSSION

### Summary

Patients or their relatives used specific phrases to express a gut feeling, indicating that they trust or do not trust the situation or perceive changes in normal, familiar patterns. Their gut feelings mostly involved a sense of alarm. Participants with a sense of alarm were convinced that something was wrong and they, particularly mothers of sick children, had often learned to trust this gut feeling. They took gut feelings about their child’s health very seriously and mentioned that they acted on them more swiftly than on gut feelings regarding their own health. When patients or their relatives could not explain symptoms, their uncertainty and anxiety increased, and they felt they were losing control. In general, a gut feeling was a reason to contact their GP to clarify the health situation, or to be reassured. Patients were less inclined to share their gut feelings with triage or practice nurses than with GPs.

Patients usually felt that primary care professionals took their gut feelings seriously.

### Strengths and limitations

To date, this is the first study exploring what phrases and expressions patients and their relatives use to voice their gut feelings, and how they share them with primary care professionals. This study included patients from the Netherlands and Belgium. The way in which patients voiced their gut feelings did not differ between participants in the two countries. It is unlikely that patients in other countries will have a different concept of gut feelings. They may use related phrases in the idiom of their language but may differ in the ways that they share their gut feelings with primary care professionals.

Fewer Belgian patients were included in the study. The interviews with the Belgian patients contained virtually no new information relating to the research questions from that of the interviews with the Dutch patients. This finding is in line with previous research results.^11^ For this reason, it was decided to stop the inclusion of any further Belgian patients.

Most members of the research team are GPs, which might have caused a limited perspective on the topic. For this reason, the input of a cognitive psychologist in the team was valuable.

### Comparison with existing literature

In a previous study that examined how patients perceived the role of GPs’ gut feelings in clinical decision making, some patients described their own gut feeling as ‘something is wrong’.^25^ They explained that their gut feeling was based on the knowledge of their body and what was normal for them. This gut feeling led to action in the form of seeking medical help. If there was mutual trust, patients mentioned their gut feeling in the consultation. These findings are in line with the results of the current study. This previous study also found that patients’ impressions of the way GPs used their own gut feelings in clinical decision making were similar to those reported by GPs.^25^

Some Belgian patients in the current study seemed to be more reticent about informing their GP of their gut feelings than Dutch patients. This might be explained by cultural differences as similar differences were found in an earlier study that explored healthcare professionals’ understanding of patients’ gut feelings.^11^ Like the current study, this previous study found no misunderstandings between GPs and patients about the notion of gut feelings.^11^ GPs indicated that they easily recognise patients’ gut feelings and consider them a useful contribution to their clinical reasoning and a better understanding of the patient’s problem.^11^

The process of developing gut feelings does not differ between GPs and patients, but the underlying knowledge and expertise does. GPs’ gut feelings are based on medical knowledge and specific expertise, while patients’ gut feelings are based on experiential knowledge about their own health and body, and may signal any changes.^1^^,^^26^ GPs’ and patients’ gut feelings are both drivers of action, such as formulating and weighing up hypotheses with a serious outcome, and calling their GP or the out-of-hours GP service, respectively.

A five-item degree-of-worry scale used in research in Denmark measures a mix of patients’ worry and gut feelings.^13^^,^^14^ The first three items ask about worry and concern, whereas the last two items describe gut feelings using phrases such as ‘a sense of urgency’, ‘a feeling of distress’, ‘the certainty that something was wrong’, and ‘a feeling of threat’. In the authors’ view, however, worry and gut feelings are different concepts. In situations where there is a clear cause, such as after a serious accident, there will be a high degree of worry or concern about the health consequences, but this will usually not lead to a gut feeling. However, in uncertain situations of illness with, in the patient’s view, unclear causes and consequences, patients might say, ‘there is something wrong with me’ or ‘it’s different from normal’, expressing their intuitive sense of alarm, which may then lead to worry or concern. Worry or concern is not based on an automatic, intuitive knowing, as is the case with a sense of alarm, but can be traced back to reasonable arguments. When composing a questionnaire for patients about their gut feelings, the difference between worry/concern and gut feelings should, therefore, be taken into account.

A GP’s sense of alarm means that the physician perceives an uneasy, intuitive feeling as they are concerned about a possible adverse outcome.^3^ It is a sense of ‘there’s something wrong here’, although specific indications have not yet been found, and this gut feeling makes the GP worried about a patient’s health situation. There is a need to initiate further diagnostics and maybe also immediate management to prevent serious health problems. However, a GP might worry about the course of the patient’s illness, for example, in the case of a patient with known serious heart failure, but still experience a sense of reassurance in the background. The physician knows how to manage the situation, for example, by prescribing effective medicines. The degree-of-worry scale described above mixes up worry/concern and gut feelings.^13^^,^^14^ In the authors’ questionnaire for GPs about their gut feelings,^4^ therefore, the word worry is only used in one item, in which the gut feeling is described as ‘something does not add up’.

### Implications for research and practice

Knowledge about the words and phrases that patients or their relatives or parents use to share their gut feelings with primary care professionals must be a vital part of professionals’ training. This will help professionals to recognise patients’ gut feelings and to understand how they come about. This study provided reliable data that was used to compose a short questionnaire to assess patients’ gut feelings, especially the more frequently mentioned sense of alarm (not yet published). This questionnaire will enable clinicians to examine the validity of patients’ gut feelings and their influence on the clinical reasoning of primary care professionals.
